# Patient-derived orthotopic xenografts of pediatric brain tumors: a St. Jude resource

**DOI:** 10.1007/s00401-020-02171-5

**Published:** 2020-06-10

**Authors:** Kyle S. Smith, Ke Xu, Kimberly S. Mercer, Frederick Boop, Paul Klimo, Michael DeCupyere, Jose Grenet, Sarah Robinson, Paige Dunphy, Suzanne J. Baker, David W. Ellison, Thomas E. Merchant, Santhosh A. Upadayaya, Amar Gajjar, Gang Wu, Brent A. Orr, Giles W. Robinson, Paul A. Northcott, Martine F. Roussel

**Affiliations:** 1 Department of Developmental Neurobiology, St. Jude Children’s Research Hospital, Memphis, TN USA; 2 Department of Computational Biology and Center for Applied Genetics, St. Jude Children’s Research Hospital, Memphis, TN USA; 3 Department of Tumor Cell Biology, St. Jude Children’s Research Hospital, Memphis, TN USA; 4 Department of Surgery, Le Bonheur Children’s Hospital, Memphis, TN USA; 5 Department of Pathology, St. Jude Children’s Research Hospital, Memphis, TN USA; 6 Department of Radiation Oncology, St. Jude Children’s Research Hospital, Memphis, TN USA; 7 Department of Oncology, St. Jude Children’s Research Hospital, Memphis, TN USA

**Keywords:** PDOX, Pediatric brain tumors, Medulloblastoma, Ependymoma, AT/RT, ETMR

## Abstract

Pediatric brain tumors are the leading cause of cancer-related death in children. Patient-derived orthotopic xenografts (PDOX) of childhood brain tumors have recently emerged as a biologically faithful vehicle for testing novel and more effective therapies. Herein, we provide the histopathological and molecular analysis of 37 novel PDOX models generated from pediatric brain tumor patients treated at St. Jude Children’s Research Hospital. Using a combination of histopathology, whole-genome and whole-exome sequencing, RNA-sequencing, and DNA methylation arrays, we demonstrate the overall fidelity and inter-tumoral molecular heterogeneity of pediatric brain tumor PDOX models. These models represent frequent as well as rare childhood brain tumor entities, including medulloblastoma, ependymoma, atypical teratoid rhabdoid tumor, and embryonal tumor with multi-layer rosettes. PDOX models will be valuable platforms for evaluating novel therapies and conducting pre-clinical trials to accelerate progress in the treatment of brain tumors in children. All described PDOX models and associated datasets can be explored using an interactive web-based portal and will be made freely available to the research community upon request.

**Electronic supplementary material:**

The online version of this article (10.1007/s00401-020-02171-5) contains supplementary material, which is available to authorized users.

## Introduction

Pediatric brain tumors are a leading cause of non-accidental death in children. Approximately, 3200 children are diagnosed with brain tumors in the United States each year; however, mortality amongst affected children is disproportionately concentrated within five malignant central nervous system (CNS) tumor entities [31]. These include high-grade gliomas (HGG), medulloblastomas (MB), ependymomas (EPN), atypical teratoid rhabdoid tumors (AT/RT), and primitive neuroectodermal tumors (PNET) [31]. PNET was a term previously used to broadly capture all embryonal tumors. This designation has recently been retired in favor of more specific terminology and from hereon, we used the updated entity designations such as embryonal tumor with multi-layer rosettes (ETMR) or CNS embryonal tumor with rhabdoid features, as appropriate [23, 42]. Unfortunately, treatment for malignant childhood CNS tumors has not radically changed over the past three decades and mostly consists of maximal safe surgical resection followed by radiotherapy (RT) with or without cytotoxic chemotherapy. Chemotherapy does not have an established role in the treatment of pediatric gliomas (i.e., HGG and EPN) and post-surgical RT is considered standard of care. In contrast, the embryonal tumors (i.e., MB, AT/RT, ETMR) are thought to be more sensitive to systemic or regionally administered chemotherapy. Patients diagnosed with embryonal tumors usually receive all three modalities, depending on age and extent of disease. Survival is non-uniform across primary tumor entities. MB and EPN are the most responsive and curable using standard approaches, while the remaining face dismal outcomes. However, even survival comes at a price, and significant treatment-associated sequelae, including moderate-to-severe neurocognitive, neuroendocrine, and psychosocial deficits, affect survivors and compromise their quality of life.

To better understand and treat these debilitating and deadly diseases, the pediatric brain tumor research community has generated a spectrum of genetically engineered mouse (GEM) models that have considerably advanced knowledge of tumor biology while providing a faithful and convenient mechanism for evaluating novel therapies [41, 43]. Unfortunately, for many entities, current GEM models do not adequately recapitulate inter-patient heterogeneity. As an example, most published GEM models of Group 3 MB (MB-G3) rely on over-expression of *MYC* or *MYCN* [17, 34, 44] and do not fully capture the heterogeneous molecular landscape that has recently been described for this subgroup [29]. In addition, GEM models of Group 4 MB (MB-G4) are mostly lacking [10], as are models of posterior fossa EPN, ETMR, and other related entities. As an alternative, patient-derived orthotopic xenografts (PDOX) [4, 13, 37, 38] of childhood brain tumors have recently emerged as an important resource for testing novel and potentially more effective therapies. Over a period of 6 years from September 2012 until March 2018, we established, characterized, and maintained 37 PDOX models representing a range of pediatric brain tumors. Several of these models have already been utilized to identify novel targeted therapies including those that have been translated into clinical trials for children with primary or recurrent/progressive malignant brain tumors [28, 40].

Here, we describe the demographic, histopathological, and molecular features of 37 PDOX models representing 5 distinct pediatric CNS tumor entities. These include tumors that are often widely prevalent in infants and children but poorly characterized and rarely studied in the laboratory. All PDOX models described in this report will be made freely available to the scientific community for conducting biological and pre-clinical studies. Such studies relying on the accessibility of faithful disease models are urgently needed to improve treatment and outcomes for childhood brain tumor patients and their families.

## Materials and methods

### PDOX model development

Tumors obtained from the day of surgery or the following morning were dissociated using the Human Tumor Dissociation Kit from Miltenyi Biotec (#130-095-929). Tumor cells were counted and implanted into the right hemisphere of 6-week-old naïve immunocompromised NodScid (NSG) mice. When tumors came from an autopsy, tumors were dissociated and implanted the next day. We implanted 2–5 mice per patient tumor with 0.2 to 1 × 10^6^ tumor cells, depending on the number of live tumor cells collected. Once growing in NSGs, passage 1 (P1), each P1 tumor was re-implanted into the right hemisphere of 5 CD1 nude (Nu/Nu) mice (P2), and each P2 tumor was amplified into 5 Nu/Nu mice to derive P3 PDOXs, without any intermediate tissue culture steps (Details provided in Supplementary Materials and methods, Online Resource).

### Tumor pathology

Histologic diagnosis of PDOX tumors and matched patient samples was assessed by hematoxylin and eosin stained section by a board-certified neuropathologist (B.A.O.) according to the criteria specified in the WHO Classification of Tumours of the Central Nervous System [23]. Immunohistochemistry was performed on 4-µm-thick formalin-fixed paraffin embedded sections using automated Ventana Benchmark or Leica Bond III machines with appropriate secondary reagents. Specific antibody clones used are listed in Supplementary Table S1, Online Resource. Dual-color FISH was performed on 4 µm paraffin embedded tissue sections. Probes were derived from BAC clones (BACPAC Resources, Oakland, CA) and labeled with either AlexaFluor-488 or AlexaFluor-555 fluorochromes (Supplementary Table S2, Online Resource). Briefly, probes were co-denatured with the target cells on a slide moat at 90 °C for 12 min. The slides were incubated overnight at 37 °C on a slide moat and then washed in 4 M Urea/2xSSC at 25 °C for 1 min. Nuclei were counterstained with DAPI (200 ng/ml; Vector Labs) for viewing on an Olympus BX51 fluorescence microscope equipped with a 100 watts mercury lamp; FITC, Rhodamine, and DAPI filters; 100× PlanApo (1.40) oil objective; and a Jai CV digital camera. Images were captured and processed using the Cytovision v7.3 software from Leica Biosystems (Richmond, IL).

### RNA and DNA extraction, library preparation, and sequencing

Genomic DNA and total RNA were simultaneously extracted from PDOXs using AllPrep DNA/RNA Mini Kit (Qiagen, Cat. #80204) following the manufacturer’s instructions. Briefly, PDOX samples were homogenized in lysis buffer using a pestle, and then disrupted tissues were transferred to a QIAshredder homogenizer column (Qiagen, Cat. #79654) and centrifuged. Lysates were transferred to an AllPrep DNA binding column. After centrifugation, the columns were kept at 4 °C for further genomic DNA purification. The eluates containing total RNA were transferred to an RNeasy column for binding total RNA by centrifugation. Columns were washed and total RNA eluted. AllPrep columns were washed and genomic DNA eluted.

RNA was quantified using the Quant-iT RiboGreen assay (Life Technologies) and quality checked by 2100 Bioanalyzer RNA 6000 Nano assay (Agilent,) 4200 TapeStation High Sensitivity RNA ScreenTape assay (Agilent,) or LabChip RNA Pico Sensitivity assay (PerkinElmer) prior to library generation. Libraries were prepared from total RNA with the TruSeq Stranded Total RNA Library Prep Kit according to the manufacturer’s instructions (Illumina, PN 20020599). Libraries were analyzed for insert size distribution on a 2100 BioAnalyzer High Sensitivity kit (Agilent Technologies,) 4200 TapeStation D1000 ScreenTape assay (Agilent Technologies,) or Caliper LabChip GX DNA High Sensitivity Reagent Kit (PerkinElmer). Libraries were quantified using the Quant-iT PicoGreen ds DNA assay (Life Technologies) or low pass sequencing with a MiSeq nano.

Genomic DNA was quantified using the Quant-iT RiboGreen assay (Life Technologies). Genomic DNA was sheared on an LE220 ultrasonicator (Covaris). Libraries were prepared from sheared DNA with HyperPrep Library Preparation Kits (Roche PN07962363001). Libraries were analyzed for insert size distribution on a 2100 BioAnalyzer High Sensitivity kit (Agilent Technologies,) 4200 TapeStation D1000 ScreenTape assay, or Caliper LabChip GX DNA High Sensitivity Reagent Kit (PerkinElmer). Libraries were quantified using the Quant-iT PicoGreen ds DNA assay (Life Technologies) or low pass sequencing with a MiSeq nano kit (Illumina). Paired-end 150 cycle sequencing was performed on a NovaSeq 6000 kit (Illumina).

### DNA methylation array analysis

All patient and PDOX samples were analyzed using either Illumina Infinium Methylation EPIC or HumanMethylation450 BeadChip arrays in accordance with manufacturer’s instructions. Briefly, genomic DNA (250–500 ng) was bisulfite treated using the Zymo EZ DNA Methylation Kit according to the following thermocycling conditions (16 cycles: 95 C for 30 s, 50 C for 1 h). Following bisulfite treatment, DNA samples were desulphonated, column purified, and then eluted using 12 ul of elution buffer (Zymo Research). Bisulfite-converted DNA (4 ul) was then processed using the Illumina Infinium Methylation Assay including hybridization to HumanMethylation850K EPIC BeadChips, single base extension assay, staining and scanning using the Illumina iScan system according to the manufacturer’s recommendations. Beta values representing the fraction of methylated cytosine present at each CpG site were calculated using the Illumina Genome Studio software using the default settings. Brain tumor entity predictions were determined using a DNA methylation-based classification web-platform for central nervous system tumors (www.molecularneuropathology.org, version 11b4) [5]. Predictions were further evaluated by implementing an ExtraTrees classifier (scikit-learn v0.20.3) trained on a reference dataset comprised of 2801 CNS tumors [29]. Resulting MB-SHH subgroup assignments were further subclassified into subtypes using another ExtraTrees classifier. The training dataset was composed of Illumina Infinium HumanMethylation450 BeadChip array data downloaded for 223 previously annotated MB-SHH samples [6]. For classification, we restricted the datasets to the 500 most informative probes based on importance scores predicted from the training cohort. Resulting entity assignments were used for all downstream analyses. Copy-number variation (CNV) analysis from methylation array data was performed with the Conumee Bioconductor package (version 1.20).

### Whole genome and whole exome sequencing analysis

Paired-end reads from tumor and germline samples were mapped to GRCh37-lite by BWA with default parameters [21]. Quality control, somatic mutation calling and classification were then conducted, as previously described [49, 50]. For PDOX samples, bam files were further processed by XenoCP for mouse read cleansing [https://www.biorxiv.org/content/10.1101/843250v3]. Reads from the original human-mapped bam file were re-aligned to the mouse reference genome using BWA. If a read’s mouse mapping score was higher than its human mapping score, the read and its mates were considered mouse reads and marked as unmapped in the cleansed bam file. Quality control, somatic mutation calling, and classifications were then conducted on the cleansed bam file and matched germline bam file. Somatic mutations occurring in protein-coding regions of signature genes were manually reviewed and reported. Germline mutations were annotated with Medal Ceremony as previously described [51]. Additional filters were applied to keep rare and potentially deleterious germline variants: (1) coverage is no less than 10×; (2) variant allele fraction (VAF) of the variant is no less than 0.2; (3) the max population frequency is less than 0.001 in ExAC; (4) non-synonymous variants including missense, nonsense, in-frame insertion/deletion (indel), frameshift indel and splice mutations; and (5) for missense mutation, REVEL score greater than 0.5 or missing REVEL scores. For tumor samples lacking paired germline samples, the variants were called by Bambino [9] and annotated by Medal Ceremony as Gold, Silver, Bronze, or Unknown [51]. We retained all the Gold variants. Qualifying non-Gold variants were also retained: (1) classified as non-silent mutations; (2) at least four mutant allele counts and at least 10x coverage; (3) at least 10% variant allele frequency; (4) minor allele frequency (MAF) < 0.01 in 1000 Genomes, MAF < 0.01 in NHLBI, and MAF < 0.001 in ExAC; (5) does not appear in more than five samples in our in-house germline mutation database; and (6) REVEL score > 0.5, if available. For paired tumor-germline samples, CNVs were detected by CONSERTING [7], and structural variants (SV) were detected by CREST [47]. Focal CNVs around genes of interest were manually inspected for changes in WGS coverage. For tumor samples without paired germline samples, we manually searched for evidence of SVs affecting gene loci by extracting the mapped reads from bam file by SAMtools and inspecting the softclip and discordant reads [22]. Oncoprint visualizations were constructed using the ComplexHeatmaps package in R [12].

### RNA sequencing analysis

Paired-end RNA-seq reads were mapped, as previously described [48]. Each read was mapped by BWA and STAR against multiple reference database files. The best alignment among different mappings was selected for inclusion in the filtered bam file. The resulting bam files from PDOX samples were further filtered by XenoCP for mouse read cleansing (https://www.biorxiv.org/content/10.1101/843250v3). We then used HTSeq-Count (version 0.11.2) to quantify the raw counts per gene [1]. Differential gene expression analyses were conducted on unbiased variance stabilizing transformed counts as implemented in DESeq 2 (v3.10) and visualized by t-distributed Stochastic Neighbor Embedding (t-SNE) [24]. Fusion genes were detected using the St. Jude CICERO pipeline, which is accessible through the St. Jude Cloud (https://platform.stjude.cloud/workflows/rapid_ma-seq).

## Results

### Development of PDOX models from malignant childhood brain tumors

**Fig. 1:**
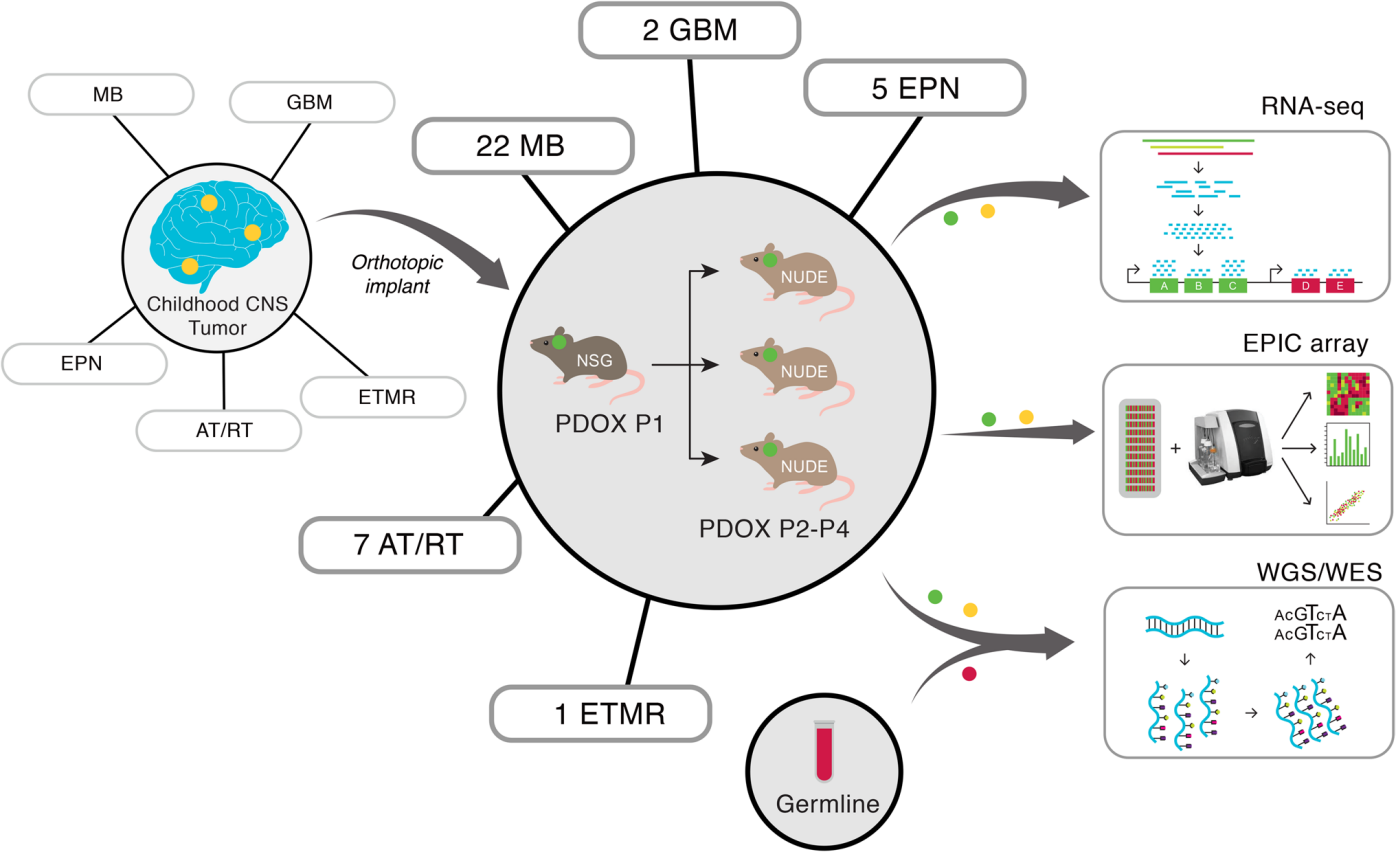
Study overview. Schematic representation of the experimental workflow used to establish and characterize 37 patient-derived orthotopic xenograft (PDOX) models from pediatric brain tumors. Patient brain tumor samples (yellow dots) implanted in immunocompromised NSG mice (P1) were subsequently transplanted into Nude mice (P2–P3) (green dots). All PDOX samples were subjected to RNA sequencing (RNA-seq), 850K DNA methylation arrays (EPIC array), whole-genome sequencing (WGS) and/or whole-exome sequencing (WES). For the 29 PDOX samples with patient-matched tumor and germline samples (red dot), both were also subjected to WGS/WES

PDOX models were derived from children diagnosed with malignant primary or recurrent brain tumors treated at St. Jude Children’s Research Hospital between 2012 and 2018 (Fig. 1). Patient tumor tissues were obtained from surgical resections performed at Le Bonheur Children’s Hospital and in some cases from autopsy. From a total of 85 patient tumor tissues implanted orthotopically into the cortices of NOD-scid IL2R-gamma (NSG) mice, 37 tumors (43% success rate overall) successfully propagated in the brains of NSG mice, each of which was further passaged in at least 5 nude mice for no more than 2–3 passages (without any intermediate tissue culture steps) in an attempt to preserve the molecular and phenotypic characteristics of the patient tumors (Table 1). Of the 37 PDOX models successfully amplified, we obtained 22 MBs (22/49, 45%) representing each of the four molecular subgroups, Wingless (MB-WNT, *n* = 3), Sonic Hedgehog (MB-SHH, *n* =8), MB-G3 (*n* =4) and MB-G4 (*n* =7); 5 EPNs (5/20, 25%) including one supratentorial RELA fusion (EPN-RELA) and four posterior fossa Group A (EPN-PFA) models; 7 AT/RTs (7/9, 77%) representing the three known molecular subgroups (AT/RT-MYC, *n* =4; AT/RT-SHH, *n* =2; AT/RT-TYR, *n* =1); and one ETMR (1/2, 50%). In addition, we established two radiation-induced HGG (i.e., glioblastoma, GBM) models (2/2, 100%) from a single patient originally diagnosed with MB-G4 (Table 1, Supplementary Table S4, Online Resource). A series of GBM and DIPG PDOX models and cell lines established from diagnostic surgical resections and biopsies will be described in a forthcoming study and will not be further detailed here (personal communication, Suzanne Baker, St. Jude Children’s Research Hospital). PDOX tumor latencies varied between 1 and 11 months (Table 1). We found that the latency of tumor growth from the time of implant to tumor harvest was remarkably unchanged from one passage to the next for all PDOXs regardless of aggressivity or molecular features. However, latency was increased by approximately 1–2 months when tumors were transplanted after cryopreservation. PDOXs were either circumscribed or difuse and metastatic (Table 1, Supplementary Fig. 1, Online Resource). We also observed the occurrence of two mouse tumors of hematopoietic origin in the brain subsequent to being implanted with human brain tumor tissue (data not shown), as previously described [4]. Early passage PDOX models were analyzed for their histopathological and molecular characteristics by whole-genome sequencing (WGS), whole-exome sequencing (WES), RNA-sequencing (RNA-seq) and EPIC DNA methylation array profiling (Fig. 1). Although not rigorously evaluated in the scope of this study, attempts to propagate various PDOX models in vitro resulted in limited success, with only a subset of tested MB, AT/RT, and EPN models showing potential compatibility for propagation in culture (data not shown).

**Table 1:** Characterization of pediatric brain tumor PDOX cohort

Sample name	PDOX identity	PDOX subtype	Patient entity	Age (years)	Sex	Gene alterations	PDOX latency (months)	Growth pattern
SJMBWNT-13-0855	MB, WNT	NA	MB, WNT	16	F	*CTNNB1*, *MLL3*	7	Circumscribed
SJMBWNT-14-5341	MB, WNT	NA	MB, WNT	9	F	*CTNNB1*, *DDX3X*	7	NA
SJMBWNT-17-00330	MB, WNT	NA	MB, WNT	5	F	*CTNNB1*, *EGFR*	5	Circumscribed
SJMBSHH -13-5634	MB, SHH	alpha	MB, SHH	6	M	*TP53*, *MYCN*	2	Diffuse
SJMBSHH -16-10230	MB, SHH	alpha	MB, SHH	6	M	*TP53*, *GLI2*	5	Diffuse
SJMBSHH -16-02525	MB, SHH	alpha	MB, SHH	9	M	*TP53*, *MYCN*,, *CDK4*, *TCF3*	4	Circumscribed
SJMBSHH -14-4106	MB, SHH	alpha	MB, SHH	8	M	*TP53*, *MYCN*, *MLL3*, *CDK4*, *TCF3*	1	Circumscribed
SJMBSHH -18-08454	MB, SHH	alpha	MB, SHH	4	M	*TP53*, *MLL2*	3	Diffuse
SJMBSHH -15-9666	MB, SHH	beta	MB, SHH	1	M	*PTCH1*	6	Circumscribed
SJMBSHH -13-6168	MB, SHH	beta	MB, SHH	3	M	*CDKN2A*	7	NA
SJMBSHH -14-7196	MB, SHH	delta	MB, SHH	8	M	*TP53*, *PTCH1*	3	Diffuse
SJMBG3-14-1830	MB, G3	I	MB, G3	3	M	*GFI1B*	11	NA
SJMBG3-12-5950	MB, G3	II	MB, G3	7	F	*MYC*	2	Diffuse
SJMBG3-16-08522	MB, G3	II	MB, G3	4	M	*MYC*, *MLL2*, *CTDNEP1*	2	Circumscribed
SJMBG3-15-10777	MB, G3	III	MB, G3	4	M	None identified	3	Circumscribed
SJMBG4-14-8531	MB, G4	V	MB, G4	13	M	*CCND2*, *CDK6*	7	Diffuse
SJMBG4-15-1259	MB, G4	VI	MB, G4	6	M	*PRDM6*, *MYCN*	9	Diffuse
SJMBG4-12-5239	MB, G4	VI	MB, G4	5	M	*KDM6A*	6	NA
SJMBG4-13-2844	MB, G4	VI	MB, G4	11	M	None identified	8	NA
SJMBG4-17-09173	MB, G4	VI	MB, G4	4	M	*TBR1*	11	Circumscribed
SJMBG4-16-08710	MB, G4	VIII	MB, G4	7	M	*MYCN*	4	Circumscribed
SJMBG4-18-03970	MB, G4	VIII	MB, G4	8	M	*MLL2*	12	Diffuse
SJGBM-14-1820	GBM, MID	NA	MB, G4	11	F	*CDKN2A/B*	2	Diffuse
SJGBM-13-3036	GBM, MID	NA	MB, G4	11	F	*CDKN2A/B*	3	Circumscribed
SJEPST-16-06903	EPN, RELA	NA	EPN, RELA	7	M	*CDKN2A/B*, *C11orf95*-*RELA*	3	Circumscribed
SJEPPF-16-02472	EPN, PFA	NA	EPN, PFA	6	M	None identified	8	Diffuse
SJEPPF-16-09238	EPN, PFA	NA	EPN, PFA	6	F	*RAG1*	7	Circumscribed
SJEPPF-15-8710	EPN, PFA	NA	EPN, PFA	7	M	None identified	7	Circumscribed
SJEPPF-16-08404	EPN, PFA	NA	EPN, PFA	8	M	*APOB*, *CDKN1B*, *CDKN2C*	4	Circumscribed
SJATRTSHH-14-3493	AT/RT, SHH	NA	AT/RT, SHH	0	F	*SMARCB1*	4	NA
SJATRTSHH-14-8191	AT/RT, SHH	NA	AT/RT, SHH	1	F	*CDKN2A*, *CDKN2A/B*, *SMARCB1*, *BRCA2*, *TSC1*	2	Circumscribed
SJATRTMYC-17-03885	AT/RT, MYC	NA	AT/RT, MYC	0	F	*SMARCB1*, *ABL1*, *NIPBL*, *ROS1*	5	Circumscribed
SJATRTMYC-17-03886	AT/RT, MYC	NA	AT/RT, MYC	0	F	*SMARCB1*, *ABL1*, *NIPBL*, *ROS1*	3	Circumscribed
SJATRTMYC-16- 03714	AT/RT, MYC	NA	AT/RT, MYC	2	M	*SMARCB1*	9	Circumscribed
SJATRTMYC-18-10115	ATRT, MYC	NA	AT/RT, SHH	0	F	*SMARCB1*	2	Circumscribed
SJATRTTYR-14-0118	AT/RT, TYR	NA	AT/RT, TYR	0	F	*SMARCB1*	10	Diffuse
SJETMR-16-07802	ETMR	NA	ETMR	2	M	*TTYH1*-*C19MC*	3	Circumscribed

Phenotypic and molecular annotations of 37 established PDOX brain tumor models. Medulloblastoma (MB), Wingless (WNT), Sonic Hedgehog (SHH), Group 3 (G3), Group 4 (G4); glioblastoma (GBM); ependymoma (EPN), supratentorial ependymoma with RELA fusion (RELA), posterior fossa ependymoma Group A (PFA); atypical teratoid rhabdoid tumor (AT/RT), Sonic Hedgehog (SHH), MYC, Tyrosinase (TYR); embryonal tumor with multi-layer rosettes (ETMR). NA=not analyzed

Forty-eight tumors (57%) failed to establish for subsequent passages, despite monitoring implanted mice for at least 12 months after the time of implantation (Supplementary Table S3, Online Resource). Of the three main tumor types included in this study (MB, EPN, and AT/RT), we had a high success rate of establishing AT/RT (77%) and lower success rate of establishing EPN (25%) models. Among MB-SHH, most of the established PDOX models harbored *TP53* mutations; whereas, those SHH tumors that did not establish harbored *PTCH1* and *ELP1* mutations. For the other entities and subgroups, we did not observe any obvious differences between the established tumors and non-established tumors. PDOXs were either circumscribed or diffuse and metastatic (Table 1, Supplementary Fig. 1, Online Resource). MB-WNT, MB-G3, and MB-G4 tumors all had a 40% success rate of establishment. *MYC* and *MYCN* amplifications were more often observed in MB-G3 and MB-G4 that were successfully established as PDOX models, but this was not statistically significant (*p* = 0.125; Fisher’s exact).

### PDOX models maintain histological features of corresponding patient tumors

**Fig. 2:**
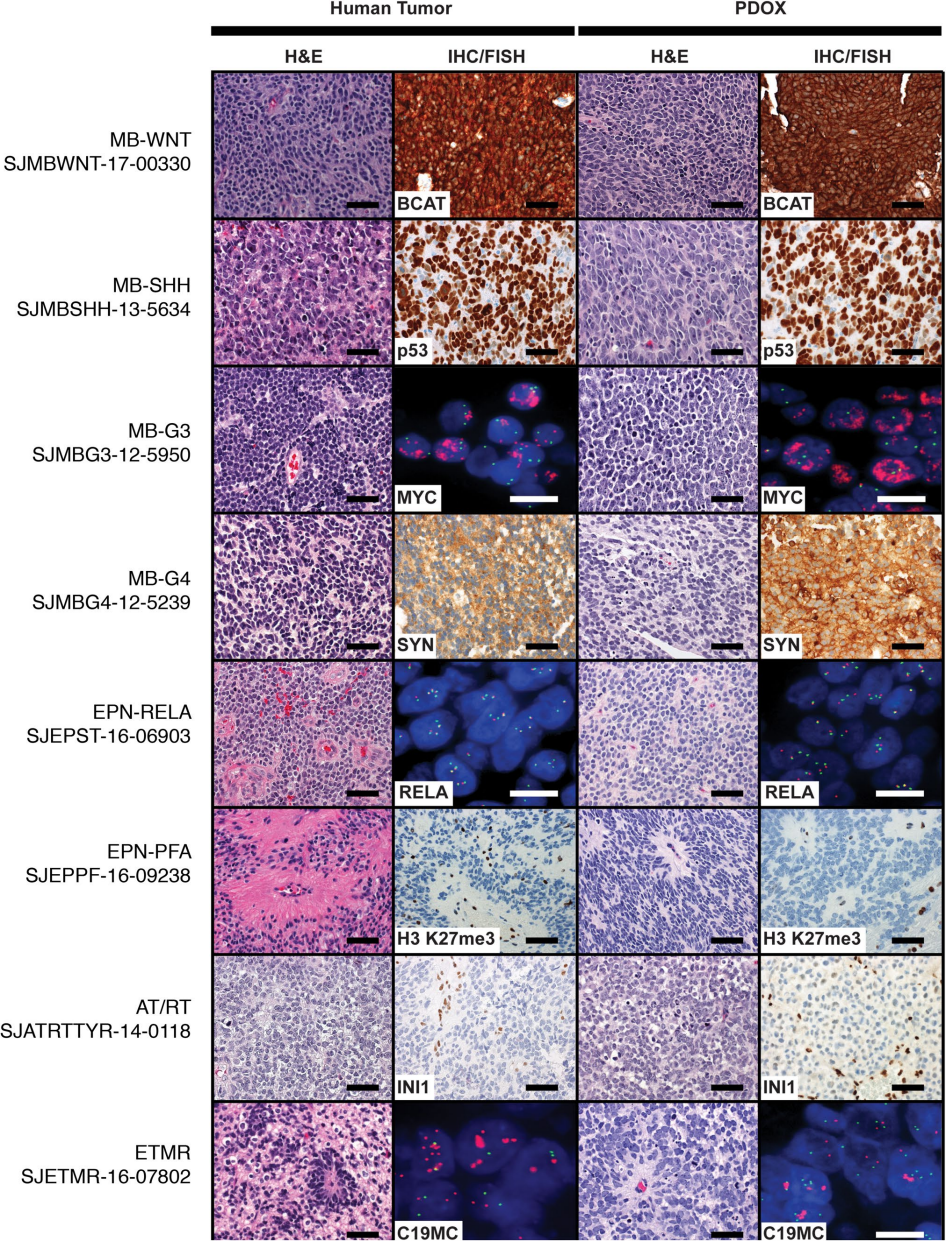
Histopathological review of PDOX tumors. Histology, immunohistochemistry (IHC), and fluorescence in situ hybridization (FISH) analysis of patient and PDOX tumors. Comparative histology of representative patient and matching PDOX tumors for each entity demonstrating stable morphology, immunophenotype, and cytogenetic features. Scale bar, 50 nM. *SYN* synaptophysin, *BCAT* β-catenin, *H3K27me3* histone 3 lysine 27 trimethylation, *L1CAM* L1 cell adhesion molecule, *C19MC* chromosome 19 microRNA cluster

Early passage PDOX tumors were evaluated histologically and compared to their corresponding patient tumors by Hematoxylin and Eosin (H&E) staining of tumor sections, by immunohistochemistry (IHC) to detect characteristic markers of each tumor entity and subgroup, and fluorescence in situ hybridization (FISH) for relevant genetic alterations (i.e., *MYC* and *MYCN* amplification in MB, C19MC amplification in ETMR). Representative examples from each childhood CNS tumor entity and their associated subgroups are shown in Fig. 2. PDOX models retained the defining histomorphology of their human tumor counterparts. For instance, MB and ETMR PDOX models all demonstrated a small cell embryonal phenotype, whereas the AT/RT PDOX models generated embryonal tumors with variable rhabdoid cells. For MB models, the classic morphology characteristic of MB-WNT was maintained in the PDOX models. Tumors showing large cell/anaplastic (LC/A) morphology in the parental tumors all demonstrated LC/A morphology in the resulting PDOXs. Slight discordance was detected in select MB PDOXs that were dominated by an anaplastic phenotype, only seen focally in the parental specimen. Likewise, there was an appreciable bias toward growth of the primitive elements of desmoplastic/nodular MB with no significant nodules of differentiation detected in the PDOX model. In EPN PDOX models, the perivascular anuclear zone (“perivascular pseudorosettes”) was appreciated in EPN-PFA (Fig. 2). Pathognomonic immunophenotypes and cytogenetic findings were also preserved in PDOX models. MB-WNT PDOX models expressed high levels of nuclear β-catenin; *TP53*-mutant MB-SHH PDOX models exhibited nuclear accumulation of TP53 protein; MB PDOX models retained expression of synaptophysin, and MB-G3 and MB-G4 PDOX models exhibited amplification of *MYC* or *MYCN* by FISH, all of which were concordant with features observed in patient-matched tumors (Fig. 2). All EPN-PFA PDOX tumors were characterized by low levels of histone 3 lysine 27 tri-methylation (H3K27me3), as previously described [15, 33]. INI1 protein expression was lost in the AT/RT PDOX models as detected by the absence of INI staining in tumor nuclei. The one ETMR PDOX model showed focal amplification of C19MC by FISH. Collectively, detailed histopathological analysis of our established PDOX models supported their fidelity to corresponding patient tumor samples exhibiting known and expected features of the respective childhood brain tumor entities included in the study cohort.

### Molecular classification and fidelity of pediatric brain tumor PDOX models

**Fig. 3:**
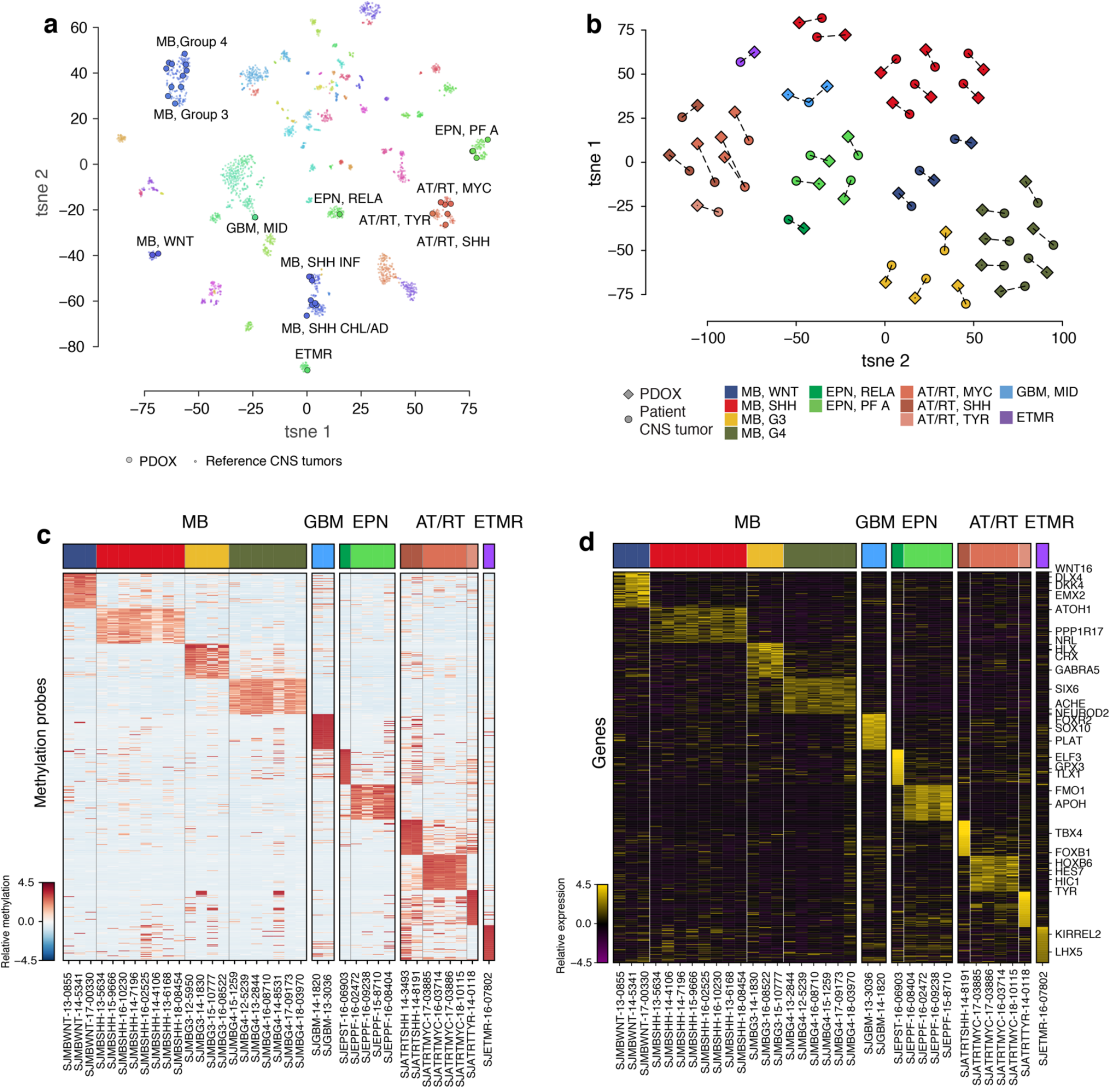
Molecular classification of PDOX models. **a** t-SNE plot of DNA methylation data for the 37 established PDOX models (black borders) alongside 2801 reference CNS tumors (no borders). **b** t-SNE plot showing pairwise DNA methylation distances of PDOX models (diamonds) and matched patient tumors (circles). **c** Unsupervised hierarchical clustering of PDOX DNA methylation array profiles based on the top 50 differentially methylated probes per subgroup/entity. Each tumor subgroup/entity is designated by a specific color. **d** Unsupervised hierarchical clustering of PDOX RNA-seq profiles based on the top 50 differentially expressed genes per tumor subgroup/entity. Each tumor subgroup/entity is designated by a specific color

Using the EPIC DNA methylation array platform, we first molecularly classified our PDOX cohort using a published Random Forest class prediction algorithm that comprises > 2800 reference CNS tumor cases and encompasses more than 100 different brain tumor entities and associated subgroups [5]. t-SNE analysis of DNA methylation profiles obtained from our PDOX models, alongside a series of annotated reference cases (*n* =2801), further substantiated molecular classification (Fig. 3a). Pairwise analysis of DNA methylation values and distances between patient-matched tumor and PDOX models showed a high degree of similarity for all models that was largely maintained amongst both early and later passage PDOX models (Fig. 3b, Supplementary Fig. 2, Online Resource). One exception pertained to a single patient diagnosed with a primary MB-G4 that yielded two radiation-induced GBM PDOX models from subsequent tumors obtained from two independent surgeries (i.e., at time of secondary malignancy and at autopsy). Cohort-wide comparison of DNA methylation signatures for the different PDOX models illustrated the overall similarity of observed methylation patterns within each of the studied CNS tumor entities and subgroups and their clear distinction from others contained in the series (Fig. 3c). Likewise, analysis of RNA-seq data from the established PDOX models (*n* =34/37) confirmed their discriminatory entity-associated expression patterns (Fig. 3d).

### Genomic landscapes of pediatric brain tumor PDOX models

For each patient tumor with matched PDOX, we performed a combination of WGS, WES, RNA-seq and EPIC DNA methylation array profiling to summarize their molecular alterations (Fig. 1). Patient germline DNA (isolated from blood) sequencing was also performed for the majority of the cohort (*n* =29/37; 78%). To further confirm that established PDOX models were genetically faithful to the patient tumors from which they were derived, we evaluated the conservation of gene-level and cytogenetic alterations amongst 35/37 models. There was overall a high degree of conservation between PDOX models and donor-matched tumor tissue profiles, especially when considering known driver gene alterations (Supplementary Figs. 3 and 4, Online Resource). Specific genomic alterations and transcriptomic signatures observed amongst the PDOX cohort are described according to brain tumor entity below.

#### MB

**Fig. 4:**
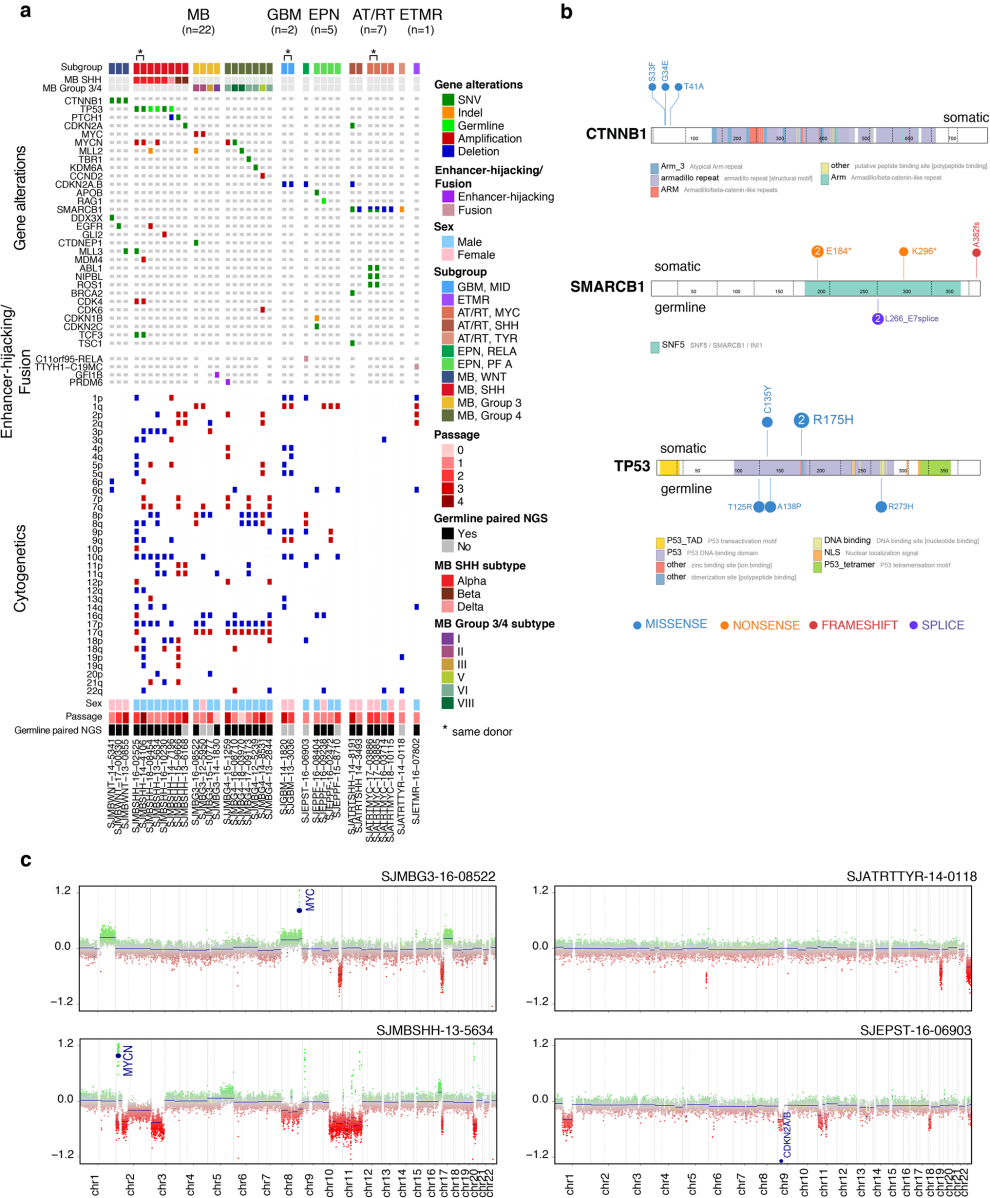
Genomic summarization of PDOX models. **a** Oncoprint highlighting gene mutations, enhancer hijacking events, gene fusions, and cytogenetic alterations observed in the PDOX cohort. **b** Examples of hallmark somatic and germline mutations detected in PDOX models. **c** Examples of chromosomal gains and losses identified in PDOX models by methylation array

A total of 22 MB-PDOX models (derived from 21 unique donors) were established, including 3 MB-WNT, 8 MB-SHH, 4 MB-G3, and 7 MB-G4 models. All three MB-WNT models harbored somatic *CTNNB1* hotspot mutations that are a defining feature of this subgroup (Fig. 4a, b). Interestingly, monosomy 6 was found in only 1 of 3 MB-WNT PDOX tumors. Additional mutations observed in MB-WNT PDOX models included *DDX3X* (*n* =1), *EGFR* (*n* =1), and *KMT2C/MLL3* (*n* =1).

Six of eight MB-SHH PDOX models (derived from 7 donors) harbored *TP53* DNA binding domain mutations (*n* =3 germline, *n* =3 somatic) that were coincident with amplification of *CDK4* (*n* =2), *MYCN* (*n* =3), or *GLI2* (*n* =1) (Fig. 4a, b). Other recurrent events amongst established MB-SHH PDOX models included somatic alterations presumed to inactivate *PTCH1* (*n* =2) or *TCF3* (*n* =2). Signature chromosomal losses of 10q (*n* =6) and 17p (*n* =5) were observed in the majority of *TP53*-mutant MB-SHH PDOX models; whereas, models harboring chromosome 9q loss were less common (*n* =3). *TP53*-mutant MB-SHH PDOX models predominantly classified as SHH-alpha tumors (*n* =5/8), consistent with what has been reported in recent literature [6, 29]. Other SHH subtypes inferred in our series included SHH-beta (*n* =2) and SHH-delta (*n* =1).

**Fig. 5:**
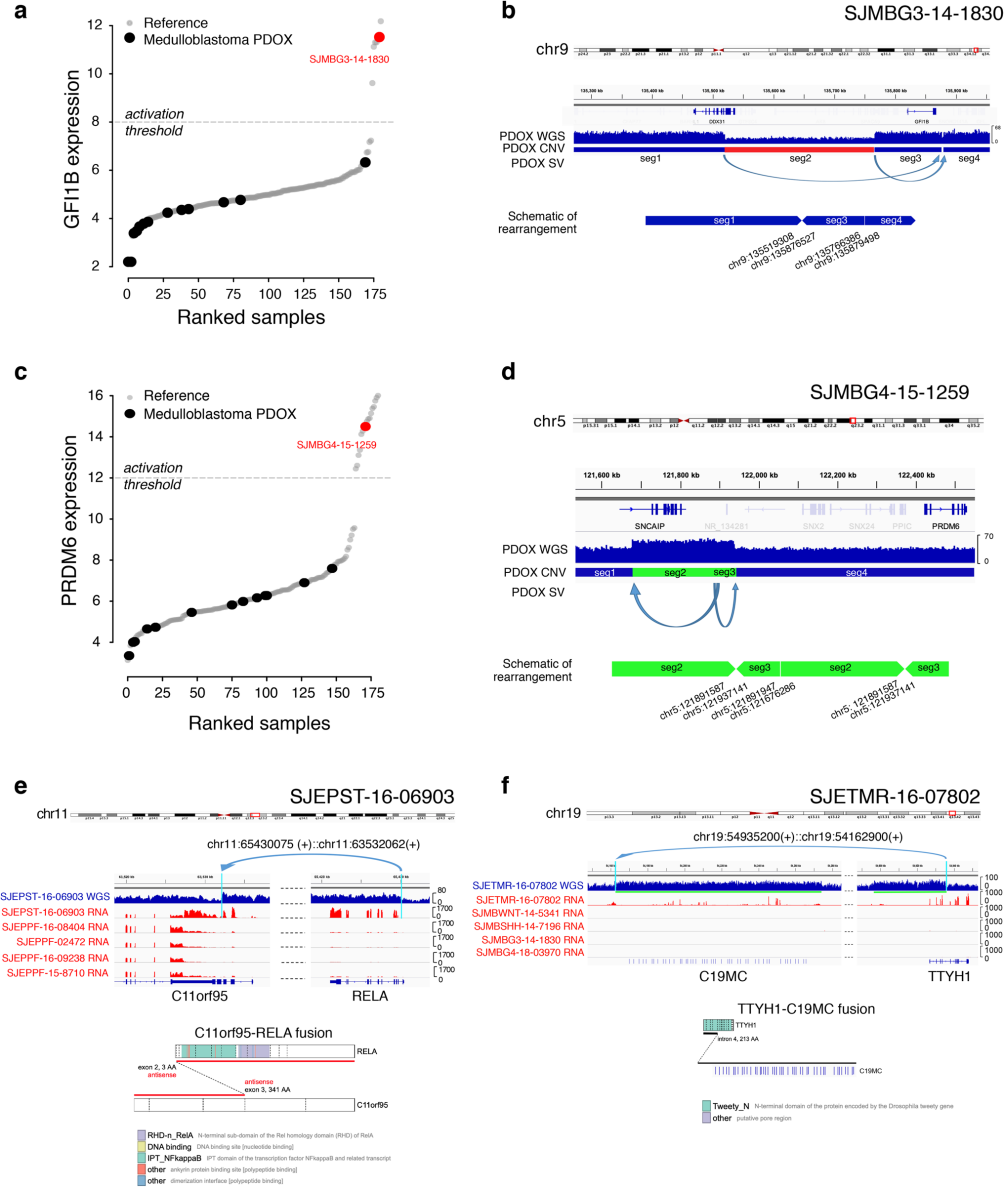
Oncogenic structural variants detected in PDOX models. **a** Over-expression of GFI1B in SJMBG3-14-1830 compared to other MB PDOX and reference MB patient samples. **b** Summary of complex structural variation detected in SJMBG3-14-1830 presumed to account for GFI1B over-expression. Red segment represents copy number loss and blue segments represent copy number neutral. **c** Over-expression of PRDM6 in SJMBG4-15-1259 compared to other MB PDOX and reference MB patient samples. **d** Summary of complex structural variation detected in SJMBG4-15-1259 presumed to account for PRDM6 over-expression. Green segments represent copy number gain and blue segments represent copy number neutral. **e** Summary of the C11orf95-RELA fusion detected in SJEPST-16-06903 and consequent high expression of RELA and C11orf95 in this PDOX compared to other EPN PDOX samples. **f** Summary of the TTYH1-C19MC fusion detected in SJETMR-16-07802 and consequent high expression of C19MC and TTYH1 in this model compared to randomly selected MB PDOX samples of each subgroup. The green lines beneath the WGS coverage track indicate single copy gain of the associated loci in the ETMR

Half (*n* =2/4) of established MB-G3 PDOX models exhibited canonical high-level *MYC* amplification (Fig. 4a, c). GFI1B over-expression associated with enhancer hijacking was observed in one MB-G3 PDOX (Figs. 4a, 5a, b) [30]. WGS analysis of the GFI1B-activated PDOX model identified prototypical structural variation on chromosome 9q34 that substantiated GFI1B over-expression (Fig. 5b). Likewise, among 7 MB-G4 PDOX models, one model exhibited PRDM6 over-expression that was supported by known enhancer hijacking-associated structural variation (Figs. 4a, 5c, d) [30]. This model also harbored high-level amplification of *MYCN*. Other notable alterations detected in MB-G4 PDOX models included mutations in chromatin modifying genes *KMT2D* (*MLL2*; *n* =1) and *KDM6A* (*n* =1), hotspot mutation in the neuronal transcription factor *TBR1* (*n* =1), and co-amplification of cell cycle genes *CCND2* and *CDK6* in a single model. The majority of MB-G3 and MB-G4 PDOX models exhibited isochromosome 17 (*n* =9/12), a signature chromosomal alteration in these subgroups. DNA methylation-based subtyping of MB-G3/G4 models identified representatives from subtypes I (*n* =1), II (*n* =2), III (*n* =1), V (*n* =1), VI (*n* =4), and VIII (*n* =2) [6, 29, 39].

#### EPN

Five EPN PDOX models were established in this series, including one EPN-RELA model and four EPN-PFA models (Fig. 4a). In addition to the *C11orf95*-*RELA fusion* that was supported at both the transcriptomic and genomic levels (Fig. 5e), the EPN-RELA model harbored homozygous deletion of the *CDKN2A/B* locus (Fig. 4a). Among the four EPN-PFA PDOX models, two had no notable mutations, one had an *APOB* mutation coupled with *CDKN1B* and *CDKN2C/p18*^*Ink4c*^ mutations, whereas the other model exhibited *RAG1* mutation. Consistent with low H3K27me3 marks, EZHIP (also known as CXorf67 and CATACOMB), was overexpressed in all EPN-PFA PDOXs (data not shown) [4, 14, 35]. Chromosome 1q gain, a hallmark feature of aggressive posterior fossa EPNs was observed in 3/4 EPN-PFA models [11, 19, 26].

#### AT/RT

AT/RT PDOX models represented each of the known molecular subgroups—SHH, MYC, and TYR—and each had either somatic mutation or focal deletion of *SMARCB1*, a hallmark feature of AT/RTs concomitant with otherwise mostly balanced genomes (Fig. 4a–c). SJATRTMYC-17-03885 and SJATRTMYC-17-03886 (derived from the same patient tumor) PDOX models exhibited simultaneous somatic mutation (located in exon 5; E184*) and focal deletion (impacting exons 2–5) of *SMARCB1* (Fig. 4a, b). Additionally, a putative pathogenic germline variant (located in exon 7; L266_E7splice) in *SMARCB1* was also detected in this patient. Whereas one AT/RT-SHH PDOX model lacked other notable mutations or chromosomal anomalies, one model harbored homozygous deletion of *CDKN2A/B* and mutations in *BRCA2* and *TSC1* (Fig. 4a).

#### ETMR

We had only a single ETMR PDOX model amongst our series and molecular characterization confirmed the presence of the *TTYH1*-*C19MC* fusion that characterizes most tumors of this rare pediatric CNS tumor entity (Figs. 4a, 5f).

### Examples of molecular discordance amongst PDOX models

Not all established PDOX tumors remained molecularly identical to the patient tumor samples from which they were derived (Supplementary Fig. 3, Online Resource). For example, among MB-SHH models, the SJMBSHH-16-02525 PDOX did not disclose the *MDM4* amplification detected in the patient tumor sample. Interestingly, the MB-G3 PDOX SJMBG3-16-08522 exhibited both *MYC* amplification and an indel in *KMT2D/MLL2* that were not detected in the primary tumor, and the SJATRTSHH-14-8191 primary tumor lacked the focal *CDKN2A/B* deletion and *BRCA2* and *TSC1* mutations that were detected in the corresponding PDOX. Equally, we found that chromosomal imbalances were not always conserved in PDOXs compared to corresponding patient tumors, observing divergence in a subset of models. Examples included the MB-WNT PDOX model, SJMBWNT-17-00330 which lacked the gain of chromosomes 5, 9, 14q, 17, and 18 that were all present in the tumor. Similarly, the MB-G3 PDOX SJMBG3-16-08522 lacked the gain of chromosomes 5 and 6 found in the primary tumor. During the molecular characterization of our PDOX cohort, two PDOX models derived from a single patient molecularly classified as GBM. The corresponding patient had been originally diagnosed with primary MB-G4 and clinically determined to have experienced ‘relapse’ of their MB. Detailed molecular analysis of the associated PDOX models determined that the patient had developed a radiation-induced secondary GBM rather than MB recurrence. Indeed, DNA methylation-based classification confirmed a high confidence molecular diagnosis of MB-G4 (MNP score for MB-G4: 0.99) for the patient’s primary tumor and GBM (MNP score for GBM-MID: 0.99) for the supposed ‘relapse’ tumor. The two PDOX models derived from this patient were established sequentially at the time of secondary malignancy and at autopsy. Comparing the methylation profiles of the molecularly diagnosed secondary GBM patient sample and the subsequent PDOX models revealed similar epigenetic profiles (Fig. 3b). The two PDOXs exhibited focal homozygous deletion of the *CDKN2A/B* locus and gain of chromosome 1q which were also confirmed in the patient’s secondary malignancy (Fig. 4a; Supplementary Fig. 3, Online Resource).

### An interactive portal for exploring pediatric brain tumor PDOX models

**Fig. 6:**
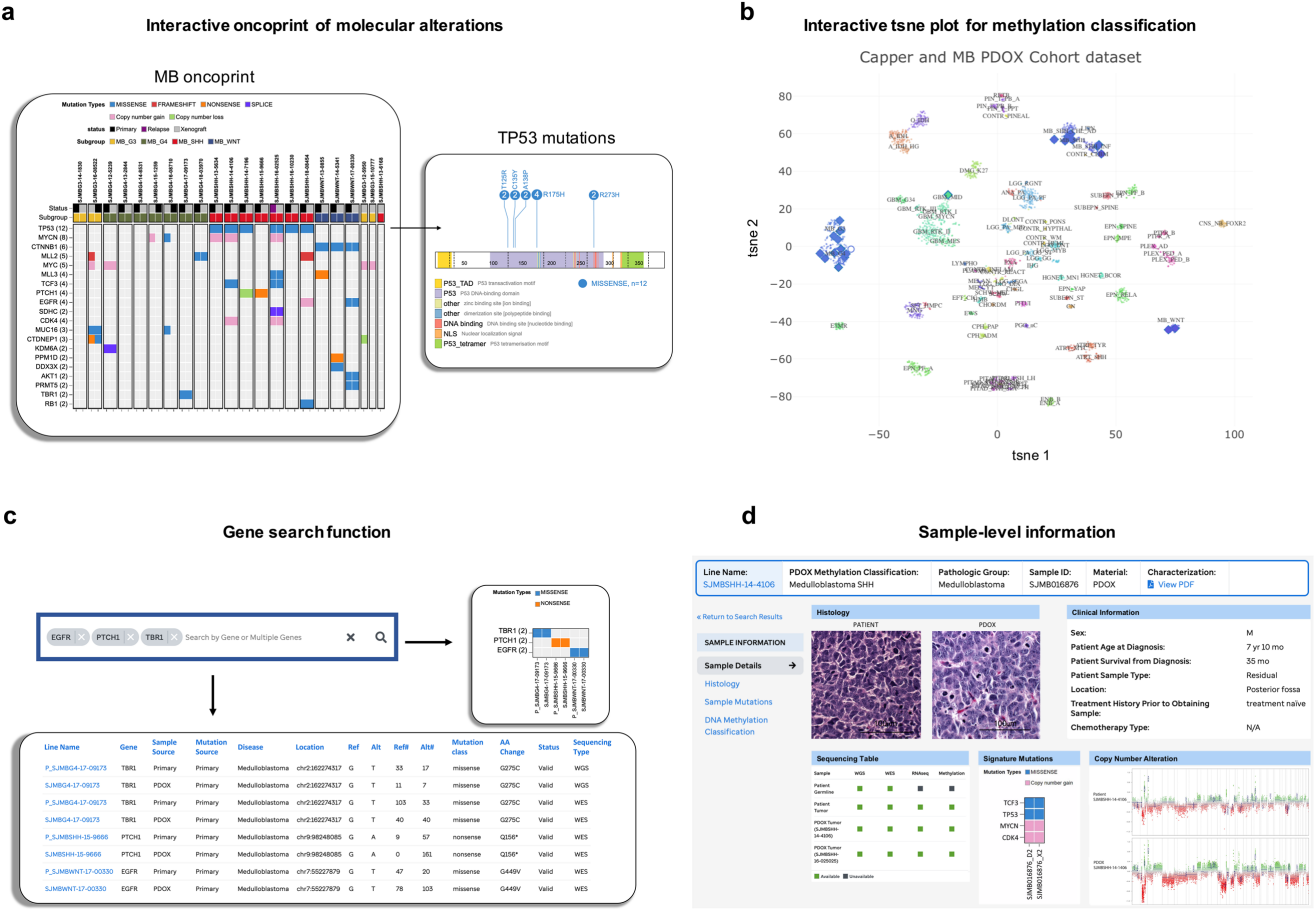
St. Jude web portal for exploring PDOX material and dataset availability, histology, molecular alterations, and methylation classification of PDOX models. **a** Screenshots of interactive oncoprints of molecular alterations in the PDOX samples and the matched patient tumor samples. Clicking on each gene can show the mutation details in affected samples. **b** A screenshot of interactive t-SNE plot for methylation classification of the MB PDOX models and the matched patient tumor samples trained on a large reference dataset of CNS tumors. Small dots are the reference tumors, larger diamonds are the PDOX samples, and larger circles are the matched patient tumor samples. **c** Screenshots of output by searching multiple genes (*EGFR, PTCH1, TBR1*) simultaneously using the gene search function. **d** Screenshots of the sample-level information for the PDOX model SJMBSHH-14-4106

We have developed a web portal (http://pbtp.stjude.cloud) to allow users to (I) interactively explore the molecular alterations in each PDOX and the matched patient tumor side-by-side (Fig. 6a); (II) interactively explore the methylation classification of each PDOX and the matched patient tumor against a large reference dataset of CNS tumors (Fig. 6b); and (III) search any gene(s) of interest and determine whether they are altered in any of the PDOX or patient samples, and explore mutation details such as amino acid position and change, mutant allele count, reference allele count, sequencing platform, and mutation flanking region (Fig. 6c). In addition to these cohort-level interactive features, users are also able to view the sample-level information including patient clinical information, PDOX sample information, availability of PDOX material, availability of raw data from sequencing and methylation array, as well as static images of histology and molecular alterations (Fig. 6d).

## Discussion

To develop new and more effective therapeutic approaches for pediatric brain tumors, laboratory models that adequately recapitulate the extensive molecular heterogeneity of these diseases are warranted. Here, we report 37 new PDOX models representing the immortalization of several pediatric brain tumor entities and their associated subgroups, including MB, AT/RT, EPN, and ETMR, many of which have no representation amongst published GEM or PDOX models. HGG PDOX models were not included here except for the radiation-induced GBM, and these will be subsequently reported with extensive preclinical testing data. As such, these PDOX models expand our stock of laboratory-available tumors and provide invaluable access to immortalized living disease specimens that can be studied and pre-clinically tested to better our understanding of these diseases and ultimately advance treatment.

We created PDOXs with 43% efficiency, irrespective of whether tumors were processed and implanted the day of surgery or early the next day. However, patient tumor samples that remained in media for ≥ 36 h failed to engraft owing to excessive cell death. We observed a wide range of tumor latencies ranging from 1 month up to 11 months, from the time of implant to the time when mice became moribund and necessitated sacrifice. Not surprisingly, the fastest growing tumors were often associated with the most clinically aggressive tumor phenotypes. AT/RT-MYC and AT/RT-SHH subgroups established efficiently and grew rapidly as did MB-SHH with *TP53* mutation and MB-G3 and MB-G4 with *MYC* or *MYCN* amplification. Typically, the most aggressive tumors grew within 1–3 months as compared to 9–11 months for other less clinically aggressive tumor subtypes (i.e., MB-WNT, *TP53*-wild type MB-SHH, MB-G4, and posterior fossa EPN) (Table 1). While other groups have reported the success of growing PDOXs in culture [4, 8, 27], we were unable to establish long-term cultures of tumor lines in vitro, using various culture conditions with the exception of one ATRT-SHH PDOX model.

Phenotypic and molecular characterization of these models based on histology, WES, WGS, RNA-seq, and DNA methylation array profiling confirmed that we generated unique and rare models. MB PDOXs included three MB-WNT models, including two without characteristic monosomy 6 and each with distinct partner mutations (i.e., *DDX3X, EGFR, MLL2*) to the common *CTNNB1* mutation; two SHH-β subtype tumors that are commonly found only in very young children [36]; two non-*MYC*-amplified MB-G3 models, one belonging to the prognostically poor subtype III category [39], and one belonging to the rare subtype I with characteristic GFI1B over-expression secondary to enhancer hijacking [30]. No genetically accurate MB-G4 GEM model currently exists and yet we established seven unique MB-G4 PDOX models including one with PRDM6 over-expression due to enhancer hijacking [29, 30], two with *MYCN* amplification, one with a *KDM6A* mutation, and one with co-amplification of *CDK6* and *CCND2*. Furthermore, we developed models from each of the three different subgroups of AT/RT (MYC, SHH, and TYR) characterized by distinct methylation signatures [16, 45, 46]; several cases of EPN-PFA with chromosome 1q gain, a well-described poor prognostic feature [11, 19, 25, 32]; and one prototypic ETMR [18, 20]. Finally, we established two secondary GBM PDOX models from a single patient with a history of MB-G4. To our knowledge, this is the only patient-derived model of a radiation-induced GBM (https://www.biorxiv.org/content/10.1101/809772v2). This diverse inventory of childhood brain tumor PDOX models illustrates their remarkable potential for future research studies to improve patient care. Such a compilation of models will enable targeted therapy testing, subgroup and subtype comparison studies, and contributes to a growing pool of tumor models that can be evaluated pre-clinically for response to targeted therapies prior to embarking on a long and often unsuccessful clinical trial that misses its target population. Whereas many of the pediatric brain tumor entities, subgroups, and genotypes have been previously established and described as PDOX models by other groups [4, 37], this St. Jude PDOX resource adds further depth to the existing collection and contributes several unique models that will be of interest to the research community. Examples include one AT/RT-TYR, two MB-G3 models without *MYC* amplification and one SHH-delta model. Models of WNT-MB, AT/RT of each subgroup, ETMR, and EPN-PFB remain rare and under-represented.

Detailed evaluation of PDOX models and their patient-matched tumor samples demonstrated a high degree of phenotypic and molecular fidelity although chromosomal imbalances were more pronounced in PDOXs than in their associated patient tumors. This corroborates a previous report in a large cohort of 1110 PDOX models across 24 cancer types, including brain tumors [2]. Here, we found that some PDOX models acquire gains of entire chromosomes or chromosome arms and in some cases gene amplifications not detected in patient tumors, whereas in other cases the opposite trend is observed. This genomic drift suggests that certain chromosomal gains and losses may be dispensable for tumorigenesis; whereas, others likely provide a selective advantage for PDOX establishment and propagation.

Despite the great potential of these new models, this study and the PDOX models themselves have their limitations. One is that a sizeable number of the patient-derived samples (47/85, 57%) failed to establish as models, and while some tumors may not have taken because of delayed time to implant, a significant number did not engraft even when implanted with abundant viable tumor cells. This suggests that some tumors may be biologically more prone to engraft than others and new methods that can increase the overall take rate need to be developed. Another drawback is that a subset of PDOX models has very long latencies, hampering their utility for pre-clinical testing that may take from several months to years to complete. Developing methods to decrease tumor latency without radically altering underlying molecular features including the development of organoids is warranted [3]. Finally, the engraftment of these tumors in immunocompromised mice creates a disingenuous environment under which these tumors grow. With all of the recent advancements in immunotherapy, efforts are especially needed to propagate these tumors in immunocompetent hosts to better explore emerging treatment options.

In conclusion, this St. Jude resource complements two previously reported cohorts of pediatric brain tumor PDOX models available to the community [4, 37] and provides a unique opportunity to conduct pre-clinical trials as a stepping stone to clinical studies. All PDOXs are available upon request using a novel St. Jude web portal, an interactive platform that will allow the scientific community to explore our datasets and request samples for their studies (Fig. 6).

## Electronic supplementary material

Below is the link to the electronic supplementary material.

Supplementary material 1 (DOCX 12717 kb)
