# Supplementary material for: Patient-derived orthotopic xenografts of pediatric brain tumors: a St. Jude resource
Source: Acta Neuropathol. 2020 Jun 10;140(2):209–25. doi: 10.1007/s00401-020-02171-5 (PMC7360541; doi:10.1007/s00401-020-02171-5)
Supplement: Supplementary file 1 — Supplementary material 1 (DOCX 12717 kb) [file 401_2020_2171_MOESM1_ESM.docx]

**Supplementary Material**

**Title: Patient-Derived Orthotopic Xenografts of Pediatric Brain Tumors: A St. Jude Resource**

**Journal: Acta Neuropathologica**

Kyle S. Smith, Ke Xu, Kimberly S. Mercer, Frederick Boop, Paul Klimo, Michael DeCupyere, Jose Grenet, Sarah Robinson, Paige Dunphy, Suzanne J. Baker, David W. Ellison, Thomas E. Merchant, Santhosh A. Upadayaya, Amar Gajjar, Gang Wu, Brent A. Orr, Giles W. Robinson, Paul A. Northcott, Martine F. Roussel

Affiliation: Department of Tumor Cell Biology, St. Jude Children’s Research Hospital, Memphis, TN, USA

Email: [martine.roussel@stjude.org](mailto:martine.roussel@stjude.org)

**Supplementary Figures**

**
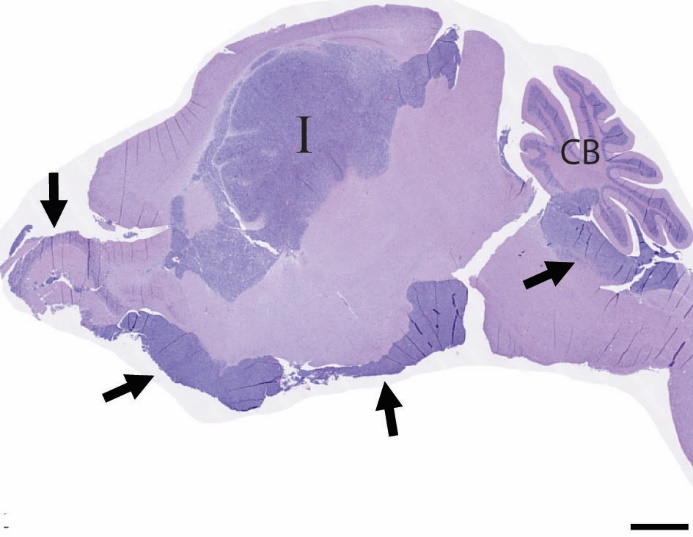
**a b


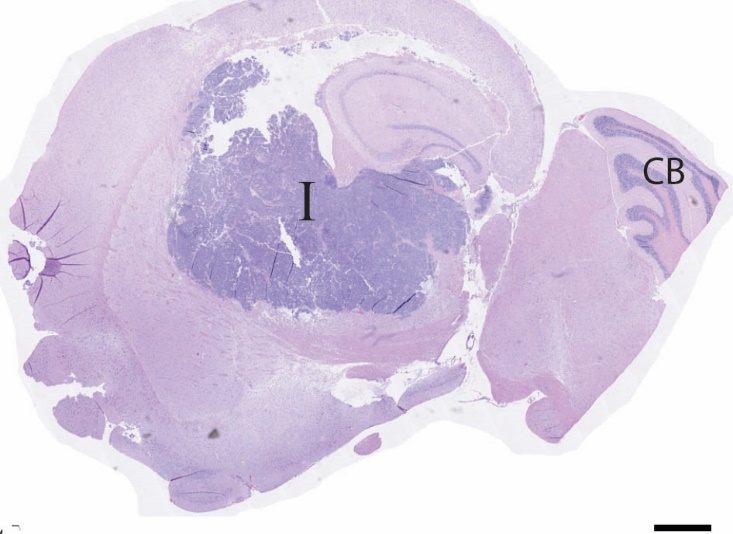


**Supplementary Fig 1. Growth pattern of two MB-G3 PDOXs.** Example of one circumscribed tumor SJMBG3-15-1077 **(a)** and one diffuse and metastatic tumor SJMBG3-12-5950 **(b)**. Black arrows indicate the sites of metastasis. Vertical black bar indicates site of implant. Horizontal black bar indicates scale bar = 1000µM.

**
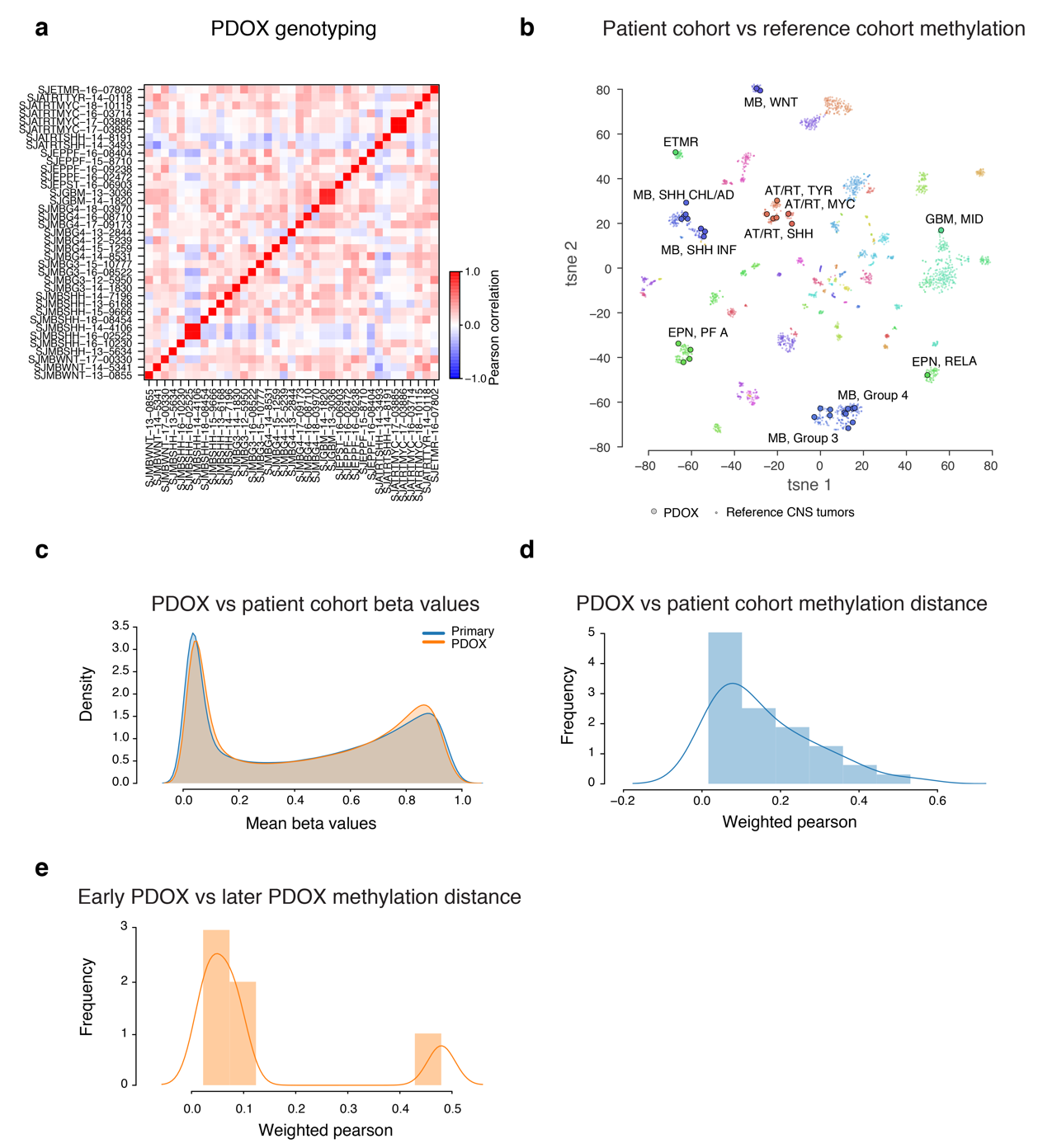
**

**Supplementary Fig 2. QC analysis of the PDOX cohort. (a)**Heatmap of PDOX Pearson correlation coefficients based on methylation beta values for genotyping probes.  **(b)** t-SNE plot of DNA methylation profiles for original patient tumor samples (filled circles) among 2,801 reference CNS tumors (dots).**(c).**Pairwise analysis of DNA methylation values between patient-matched tumor and PDOX models. **(d)** Pairwise analysis of DNA methylation distances between patient-matched tumor and PDOX models. **(e)** Pairwise analysis of DNA methylation distances between select early and late passage and PDOX models.

**
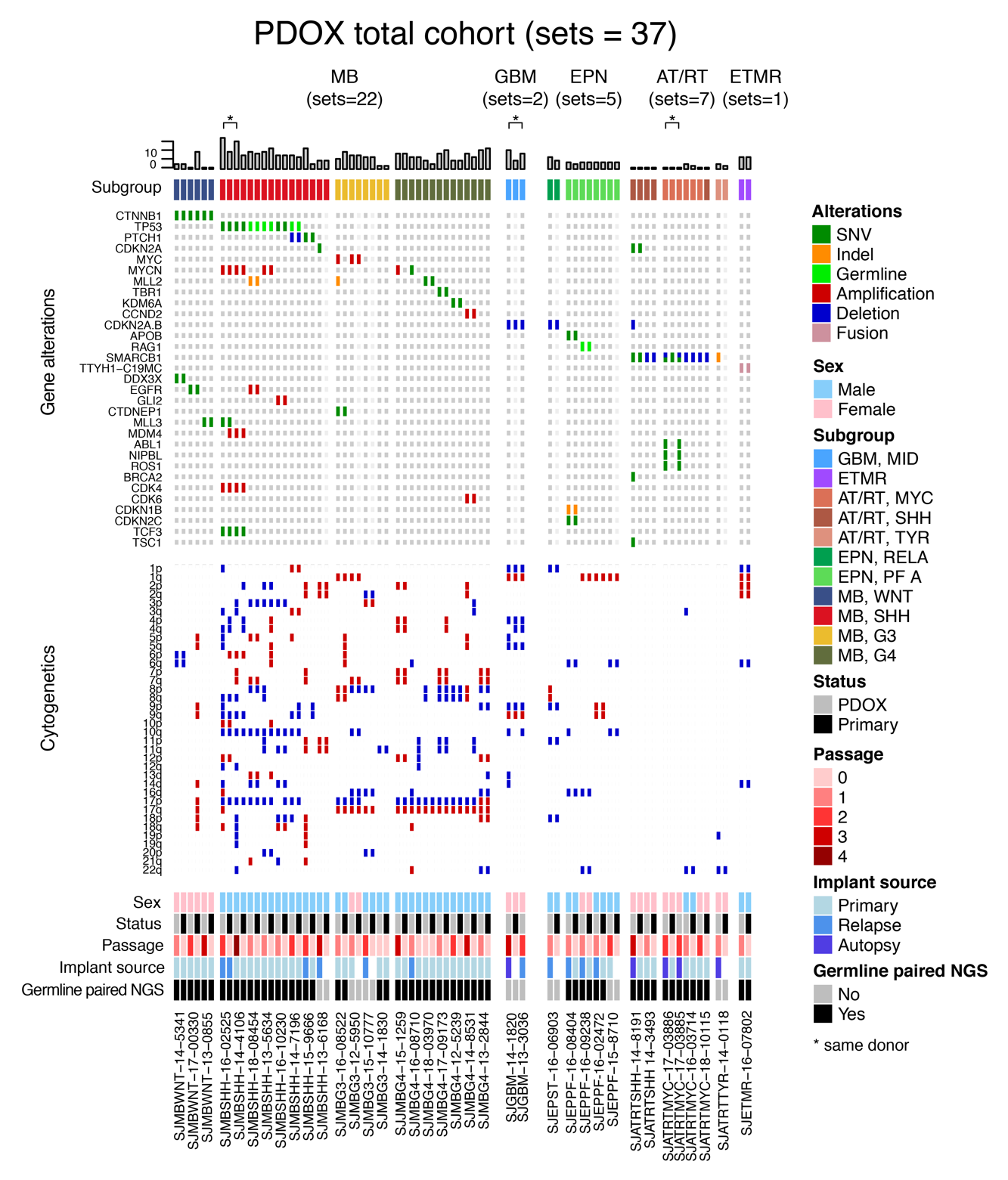
**

**Supplementary Fig 3. Molecular features of the PDOX model cohort.** Oncoprint summarizing the molecular features and metadata for the patient-matched tumor and PDOX cohort.

**
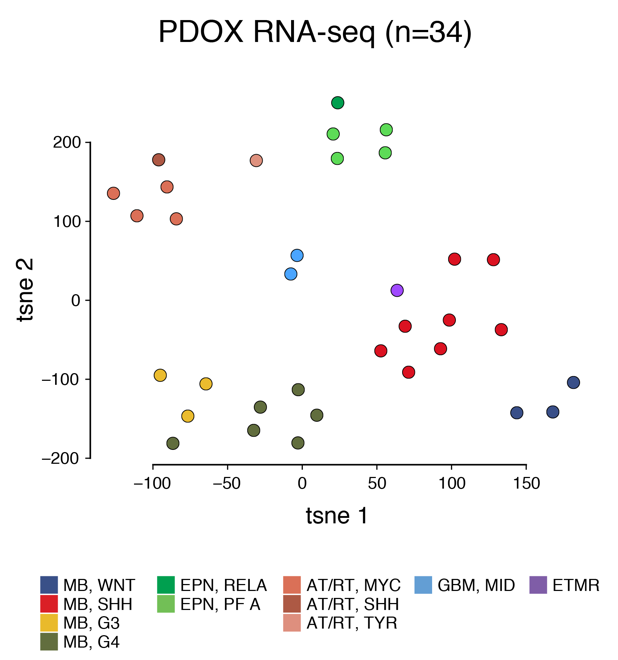
**

**Supplementary Fig 4. PDOX RNA-seq dataset.** t-SNE plot of RNA-seq data for PDOX models with available RNA-seq data (n=34).

**Supplementary Tables**

**Table S1. Antibodies used for immunohistochemistry.**

| **Target** | **Clone** | **Catalog Number** | **Company** | **Dilution** |
| --- | --- | --- | --- | --- |
| Synaptophysin | 27G2 | NCL-L-SYNAP-299 | Leica Microsystems | 1:200 |
| GFAP | 6F2 | MO76101 | Dako | 1:400 |
| Olig2 | EP112 | 790-4509 | Ventana | Ready to use |
| YAP1 | 63.7 | SC-101199 | Santa Cruz | 1:50 |
| GAB1 | H-7 | SC-133191 | Santa Cruz | 1:800 |
| Β-Catenin | Clone 14 | 760-4242 | Ventana | Ready to use |
| L1CAM | UJ127.11 | L4543 | Sigma Aldrich | Ready to use |
| INI-1 | MRQ-27 | 272M-15 | Cell Marque | Ready to use |
| P53 | DO-7 | Z2029M | Zeta Corp | 1:200 |
| H3K27-trimethylation | C36B11 | 9733 | Cell Signaling | Ready to use |

**Table S2. Probes used for fluorescence in situ hybridization (FISH).**

| **Probe** | **Clone** | **Locus** |
| --- | --- | --- |
| 1q | RP11-610024 | 1q43 |
| 1q control | CTD-3241G19 | 1p13.1 |
| *MYCN* probe 1 | RP11-355H10 | 2p24.1 |
| *MYCN* probe 2 | RP11-348M12 | 2p24.1 |
| *MYCN* control 1 | RP11-296A19 | 2q35 |
| *MYCN* control 2 | RP11-38408 | 2q35 |
| *PDGFRA* probe 1 | RP11-231C18 | 4q12 |
| *PDGFRA* probe 2 | RP11-601I15 | 4q12 |
| *PDGFRA* control 1 | CTD-2057N12 | 4q12 |
| *PDGFRA* control 2 | CTD-2588A19 | 4q12 |
| *MYC* probe 1 | CTD-3056022 | 8q24 |
| *MYC* probe 2 | CTD-2267H22 | 8q24 |
| *MYC* control 1 | RP11-1077A8 | 8p11.21 |
| *MYC* control 2 | RP11-867P15 | 8p11.21 |
| *CDKN2A* probe 1 | RP11-14912 | 9p21.3 |
| *CDKN2A* probe 2 | RP11-145E5 | 9p21.3 |
| *CDKN2A* control | RP11-235C23 | 9q31.2 |
| *PTCH1* probe 1 | RP11-150G22 | 9q22.32 |
| *PTCH1* control 1 | RP11-643P16 | 9p21.1 |
| *PTCH1* control 2 | RP11-45J11 | 9p21.1 |
| *C11orf95_BAP_1A* | CH17-215P06 | 11q13.1 |
| *C11orf95_BAP_1B* | CH17-67K13 | 11q13.1 |
| *C11orf95_BAP_2* | CH17-388O01 | 11q13.1 |
| *RELA_*BAP_1A | CH17-14J18 | 11q13.1 |
| *RELA_*BAP_1B | RP11-641F7 | 11q13.1 |
| *RELA_*BAP_2 | CH17-211O12 | 11q13.1 |
| *C19MC* probe | RP11-948E8 | 19q13.4 |
| *C19MC* control 1 | CTD-2538G9 | 19p13.11 |
| *C19MC* control 2 | CTD-2528A14 | 19p13.11 |

**Table S3. Implanted pediatric brain tumor samples that did not engraft as PDOX**. Medulloblastoma (MB), Wingless (WNT), Sonic Hedgehog (SHH), Group 3 (G3), Group 4 (G4); supratentorial ependymoma with RELA fusion (EPN, RELA), supratentorial ependymoma (EPN, ST), supratentorial ependymoma with YAP1 fusion (EPN, YAP1), posterior fossa ependymoma Group A (EPN, PFA), myxopapillary ependymoma (EPN, MYX); atypical teratoid rhabdoid tumor (AT/RT); embryonal tumor with multi-layer rosettes (ETMR); pineoblastoma (PB); pineal parenchymal tumor with intermediate differentiation (PPTID); high-grade neuroepithelial tumor (HGNET). Not otherwise specified (NOS).

| Sample Name | Patient tumor identity | Patient tumor subtype | Age  (years) | Sex | Tumor Characteristics |
| --- | --- | --- | --- | --- | --- |
| SJMBWNT-13-2911 | MB, WNT | NA | 5 | F | Monosomy 6 |
| SJMBWNT-14-6507 | MB, WNT | NA | 5 | M | Monosomy 6 |
| SJMBWNT-15-1928 | MB, WNT | NA | 16 | F | Monosomy 6 |
| SJMBWNT-17-01099 | MB, WNT | NA | 17 | M | Monosomy 6 |
| SJMBSHH-13-2805 | MB, SHH | alpha | 4 | F | *PTCH1*, *ELP1* |
| SJMBSHH-13-3258 | MB, SHH | alpha | 5 | F | *PTCH1*, *ELP1* |
| SJMBSHH-14-3384 | MB, SHH | alpha | 11 | M | *PTCH1* |
| SJMBSHH-17-10449 | MB, SHH | alpha | 4 | F | *PTCH1, ELP1* |
| SJMBSHH-17-03577 | MB, SHH | delta | 13 | M | *PTCH1* |
| SJMBSHH-15-9739 | MB, SHH | unknown | 1 | M | *PTCH1* |
| SJMBG3-15-0937 | MB, G3 | I | 4 | M | None identified |
| SJMBG3-17-02763 | MB, G3 | II | 11 | F | i17q |
| SJMBG3-17-03618 | MB, G3 | II | 5 | M | None identified |
| SJMBG3-16-00266 | MB, G3 | III | 3 | M | *PTEN* |
| SJMBG3-13-6624 | MB, G3 | IV | 7 | M | None identified |
| SJMBG3-16-02453 | MB, G3 | IV | 2 | F | Hyperdiploid |
| SJMBG4-14-5074 | MB, G4 | I | 12 | M | None identified |
| SJMBG4-16-10856 | MB, G4 | V | 11 | M | i17q |
| SJMBG4-15-0158 | MB, G4 | VII | 7 | M | None identified |
| SJMBG4-15-7001 | MB, G4 | VII | 11 | M | *MYCN* amp, i17q |
| SJMBG4-14-1748 | MB, G4 | VII | 12 | M | None identified |
| SJMBG4-13-5981 | MB, G4 | VIII | 8 | M | None identified |
| SJMBG4-13-6391 | MB, G4 | VIII | 8 | M | None identified |
| SJMBG4-14-8153 | MB, G4 | VIII | 17 | M | None identified |
| SJMBG4-15-7003 | MB, G4 | VIII | 7 | M | i17q |
| SJMBG4-17-11625 | MB, G4 | VIII | 9 | F | *SETD2* |
| SJMBNOS-13-0447 | MB, NOS | NA | 3 | M | None identified |
| SJMBNOS-16-01785 | MB, NOS | NA | 17 | M | None identified |
| SJEPST-16-08211 | EPN, RELA | NA | 8 | M | *RELA* fusion |
| SJEPST-16-08614 | EPN, RELA | NA | 4 | M | *RELA* fusion |
| SJEPST-17-04700 | EPN, RELA | NA | 16 | M | *RELA* fusion |
| SJEPST-17-02192 | EPN, ST | NA | 15 | M | No *RELA* or *YAP1* |
| SJEPST-17-01068 | EPN, ST | NA | 17 | M | 1q gain, no *RELA* or *YAP1* |
| SJEPST-15-10010 | EPN, YAP1 | NA | 2 | F | *YAP1-MAMLD1* |
| SJEPPF-15-9254 | EPN, PFA | NA | 10 | F | No 1q gain |
| SJEPPF-15-9759 | EPN, PFA | NA | 1 | M | No 1q gain |
| SJEPPF-16-07399 | EPN, PFA | NA | 5 | F | No 1q gain, *BCOR* |
| SJEPPF-16-07964 | EPN, PFA | NA | 2 | F | No 1q gain |
| SJEPPF-16-08731 | EPN, PFA | NA | 3 | M | 1q gain |
| SJEPPF-17-00535 | EPN, PFA | NA | 18 | M | 1q gain |
| SJEPPF-17-07683 | EPN, PFA | NA | 2 | M | 1q gain |
| SJEPMYX-16-09447 | EPN, MYX | NA | 15 | M | None identified |
| SJATRT-15-4717 | AT/RT, NOS | NA | 2 | M | 22q11.23 deletion syndrome |
| SJATRT-17-03859 | AT/RT, NOS | NA | 3 | M | *SMARCB1*, *VHL* |
| SJETMR-13-6615 | ETMR | NA | 3 | F | *C19MC* AMP |
| SJPB-17-00879 | PB | NA | 1 | M | None identified |
| SJPPTID-14-7309 | PPTID | NA | 7 | F | None identified |
| SJHGNET-17-10570 | HGNET | NA | 10 | F | *DICER*, *KRAS* |

**Table S4. PDOX classification.** Classifier outputs for all samples of the PDOX cohort (37 models) and matched patient samples (n=35).

| **Sample name** | **PDOX MNP** | **Patient MNP** | **PDOX ExtraTrees** | **Patient ExtraTrees** |
| --- | --- | --- | --- | --- |
| SJMBWNT-13-0855 | MB_SHH_CHL_AD | MB_SHH_CHL_AD | MB_SHH_CHL_AD | MB_SHH_INF |
| SJMBWNT-14-5341 | MB_WNT | MB_WNT | MB_WNT | MB_WNT |
| SJMBWNT-17-00330 | MB_WNT | MB_WNT | MB_WNT | MB_WNT |
| SJMBSHH-13-5634 | MB_SHH_CHL_AD | MB_SHH_CHL_AD | MB_SHH_CHL_AD | MB_SHH_CHL_AD |
| SJMBSHH-16-10230 | MB_SHH_CHL_AD | MB_SHH_CHL_AD | MB_SHH_CHL_AD | MB_SHH_CHL_AD |
| SJMBSHH-16-02525 | PLEX_PED_B | PLEX_PED_B | MB_SHH_CHL_AD | MB_SHH_CHL_AD |
| SJMBSHH-14-4106 | PLEX_PED_B | PLEX_PED_B | MB_SHH_CHL_AD | MB_SHH_CHL_AD |
| SJMBSHH-18-08454 | MB_SHH_CHL_AD | MB_SHH_INF | MB_SHH_CHL_AD | MB_SHH_CHL_AD |
| SJMBSHH-15-9666 | MB_SHH_INF | MB_SHH_INF | MB_SHH_INF | MB_SHH_INF |
| SJMBSHH-13-6168 | MB_SHH_INF | MB_SHH_INF | MB_SHH_INF | MB_SHH_INF |
| SJMBSHH-14-7196 | MB_SHH_CHL_AD | MB_SHH_CHL_AD | MB_SHH_CHL_AD | MB_SHH_CHL_AD |
| SJMBG3-14-1830 | MB_G3 | MB_G3 | MB_G3 | MB_G3 |
| SJMBG3-12-5950 | MB_G3 | MB_G3 | MB_G3 | MB_G3 |
| SJMBG3-16-08522 | MB_G3 | MB_G3 | MB_G3 | MB_G3 |
| SJMBG3-15-10777 | MB_G3 | MB_G3 | MB_G3 | MB_G3 |
| SJMBG4-14-8531 | MB_G4 | MB_G4 | MB_G4 | MB_G4 |
| SJJMBG4-15-1259 | MB_G4 | MB_G4 | MB_G4 | MB_G4 |
| SJMBG4-12-5239 | MB_G4 | MB_G4 | MB_G4 | MB_G4 |
| SJMBG4-13-2844 | MB_G4 | MB_G4 | MB_G4 | MB_G4 |
| SJMBG4-17-09173 | MB_G4 | MB_G4 | MB_G4 | MB_G4 |
| SJMBG4-16-08710 | MB_G4 | MB_G4 | MB_G4 | MB_G4 |
| SJMBG4-18-03970 | MB_G4 | MB_G4 | MB_G4 | MB_G4 |
| SJGBM-14-1820 | None | MB_G4 | GBM_MID | MB_G4 |
| SJGBM-13-3036 | GBM_MID | MB_G4 | GBM_MID | MB_G4 |
| SJEPST-16-06903 | EPN_RELA | EPN_RELA | EPN_RELA | EPN_RELA |
| SJEPPF-16-02472 | EPN_PF_A | EPN_PF_A | EPN_PF_A | EPN_PF_A |
| SJEPPF-16-09238 | EPN_PF_A | EPN_PF_A | EPN_PF_A | EPN_PF_A |
| SJEPPF-15-8710 | EPN_PF_A | EPN_PF_A | EPN_PF_A | EPN_PF_A |
| SJEPPF-16-08404 | EPN_PF_A | EPN_PF_A | EPN_PF_A | EPN_PF_A |
| SJATRTSHH 14-3493 | ATRT_SHH | ATRT_SHH | ATRT_SHH | ATRT_SHH |
| SJATRTSHH-14-8191 | ATRT_SHH | ATRT_SHH | ATRT_SHH | ATRT_SHH |
| SJATRTMYC-17-03885 | ATRT_MYC | ATRT_MYC | ATRT_MYC | ATRT_MYC |
| SJATRTMYC-16-03714 | ATRT_MYC | ATRT_MYC | ATRT_MYC | ATRT_MYC |
| SJATRTMYC-17-03886 | ATRT_MYC | ATRT_MYC | ATRT_MYC | ATRT_MYC |
| SJATRTMYC-18-10115 | ATRT_MYC | ATRT_SHH | ATRT_MYC | ATRT_SHH |
| SJATRTTYR-14-0118 | ATRT_TYR | ATRT_TYR | ATRT_TYR | ATRT_TYR |
| SJETMR-16-07802 | ETMR | ETMR | ETMR | ETMR |

**Supplementary Materials and Methods**

**Development of patient-derived orthotopic xenografts**

Tumor samples received the day of surgery or the next morning and stored in neurobasal medium overnight (O/N) were dissociated using the Human Tumor Dissociation Kit from Miltenyi Biotec (#130-095-929).  Briefly, tumor tissue was cut into 2mm size pieces using a sterile scalpel blade, transferred to into a 15mL falcon tube containing the enzyme mixture.  Cells were dissociated with a 5mL serologic pipette and incubated at a water bath ar 37^0^C degrees for 30 minutes, agitating the tube every 10 minutes.  Tumor cells were passed through a 70uM cell strainer with 10mL neurobasal media.  The sample was centrifuged for 7 minutes at 300g, the supernatant was discarded, and dissociated cells treated with one mL of Red Cell Lysis Buffer from Sigma (R7757) for 1 minute.  Ten mLs of neurobasal media was added to tumor cells and cells centrifuged, 7 minutes at 300g.  The supernatant was discarded, tumor cells resuspended into neurobasal media and counted. 0.2 to 1 x 10^6^ tumor cells in 5 ul of matrigel per animal were implanted into the right hemisphere of 6-week- old naïve NodScid (NSG) mice.  Before performing implants, mice were given a combination of xylazine and ketamine via intraperitoneal injection to maintain a surgical plane of anesthesia for 30-40 minutes.  Sedated mice were placed on a stereotax and secured.    An incision was made to the right of the midline of the scalp.  The fascia was removed, and the skin pushed aside using a sterile cotton tip applicator.  A dental drill was used to cut a small square section of the skull cap.  The square was removed using a pair of Dumont forceps.  The area was flushed with sterile saline, and light suction was used to remove the saline and remaining bone dust.  Using a blunt Hamilton syringe, 5uL of tumor cells with matrigel were injected into the right hemisphere.  Once the injection was complete, the animal was removed from the stereotactic instrument and the incision site closed using two wound clips. Mice were placed in a clean, prewarmed cage until fully recovered.  Mice remained on heat for 48 hours post-surgery.  Wound clips were removed 7-10 days post op.  Once tumors grew in NSGs, passage 1 (P1), each tumor was re-implanted into the cortices of 5 nude Nu/Nu mice (P2), and each P2 tumor amplified into 5 Nude Nu/Nu mice to derive P3 PDOXs.   NSG mice were used in the primary implant to increase the chance of engraftment. However, once tumors are established in NSG mice, they always subsequently grow in CD1 nude mice. In addition, CD1 nude mice were used for subsequent passages because they are less prone to infection than NSG mice and thus easier to maintain and use in pre-clinical trials. It is very possible that the primary human tumors could engraft in CD1 nude mice, but we never have enough primary human tumor tissue to test this possibility.
